# Large‐vessel vasculitis induced by pegfilgrastim and immune checkpoint inhibitor in a patient with small‐cell lung cancer

**DOI:** 10.1002/rcr2.1291

**Published:** 2024-02-07

**Authors:** Keisuke Shiraha, Yuki Takigawa, Akiko Sato, Keiichi Fujiwara, Yuka Matsuo, Mayu Goda, Tomoyoshi Inoue, Eri Nakamura, Miho Fujiwara, Suzuka Matsuoka, Hiromi Watanabe, Kenichiro Kudo, Ken Sato, Takuo Shibayama

**Affiliations:** ^1^ Department of Respiratory Medicine NHO Okayama Medical Center Okayama Japan

**Keywords:** immune checkpoint inhibitor, immune‐related adverse event, large‐vessel vasculitis, pegfilgrastim, small‐cell lung cancer

## Abstract

A 75‐year‐old woman with stage IVB (cT3N3M1c) extensive disease small‐cell lung cancer was treated with carboplatin, etoposide, and atezolizumab. Ten days after pegfilgrastim initiation, during the second chemotherapy cycle, she experienced back pain. Contrast‐enhanced computed tomography revealed soft tissue thickening around the descending aorta and brachiocephalic artery. She was diagnosed with atezolizumab and pegfilgrastim‐induced large‐vessel vasculitis (LVV) and was treated with prednisolone, which was tapered and discontinued after 14 weeks, with no symptom recurrence. LVV should be included in the differential diagnosis of patients with nonspecific body pain when pegfilgrastim and immune checkpoint inhibitors are used in combination.

## Graphical abstract

The present case was diagnosed with atezolizumab and pegfilgrastim‐induced large‐vessel vasculitis (LVV) and was treated with prednisolone. LVV should be included in the differential diagnosis of patients with nonspecific body pain when pegfilgrastim and immune checkpoint inhibitors are used in combination.

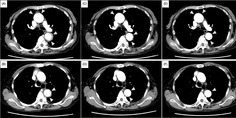

## INTRODUCTION

Granulocyte‐colony stimulating factor (G‐CSF) is widely used to manage malignant tumours to prevent chemotherapy‐induced febrile neutropaenia (FN). Large‐vessel vasculitis (LVV) is an adverse event of G‐CSF.^1^ Pegfilgrastim is a pegylated long‐acting recombinant form of G‐CSF that causes frequent LVV.^1^ Immune checkpoint inhibitors (ICIs) causing LVV have also been reported.^2^

We report a rare case of LVV possibly induced by pegfilgrastim and ICI during treatment with atezolizumab in a patient with extensive disease small‐cell lung cancer (ED‐SCLC).

## CASE REPORT

A 75‐year‐old woman was referred to our hospital with severe dyspnoea and abnormal liver function. Computed tomography (CT) revealed a lung mass and liver metastasis; thus, the patient underwent bronchoscopy and was diagnosed with stage IVB (cT3N3M1c) ED‐SCLC. She was treated with the first cycle of chemotherapy consisting of carboplatin, etoposide, and atezolizumab,^3^ and developed FN. To prevent myelosuppression and FN, the second cycle of chemotherapy was administered at a reduced dose, and pegfilgrastim was administered 15 days before emergency admission.

**FIGURE 1:**
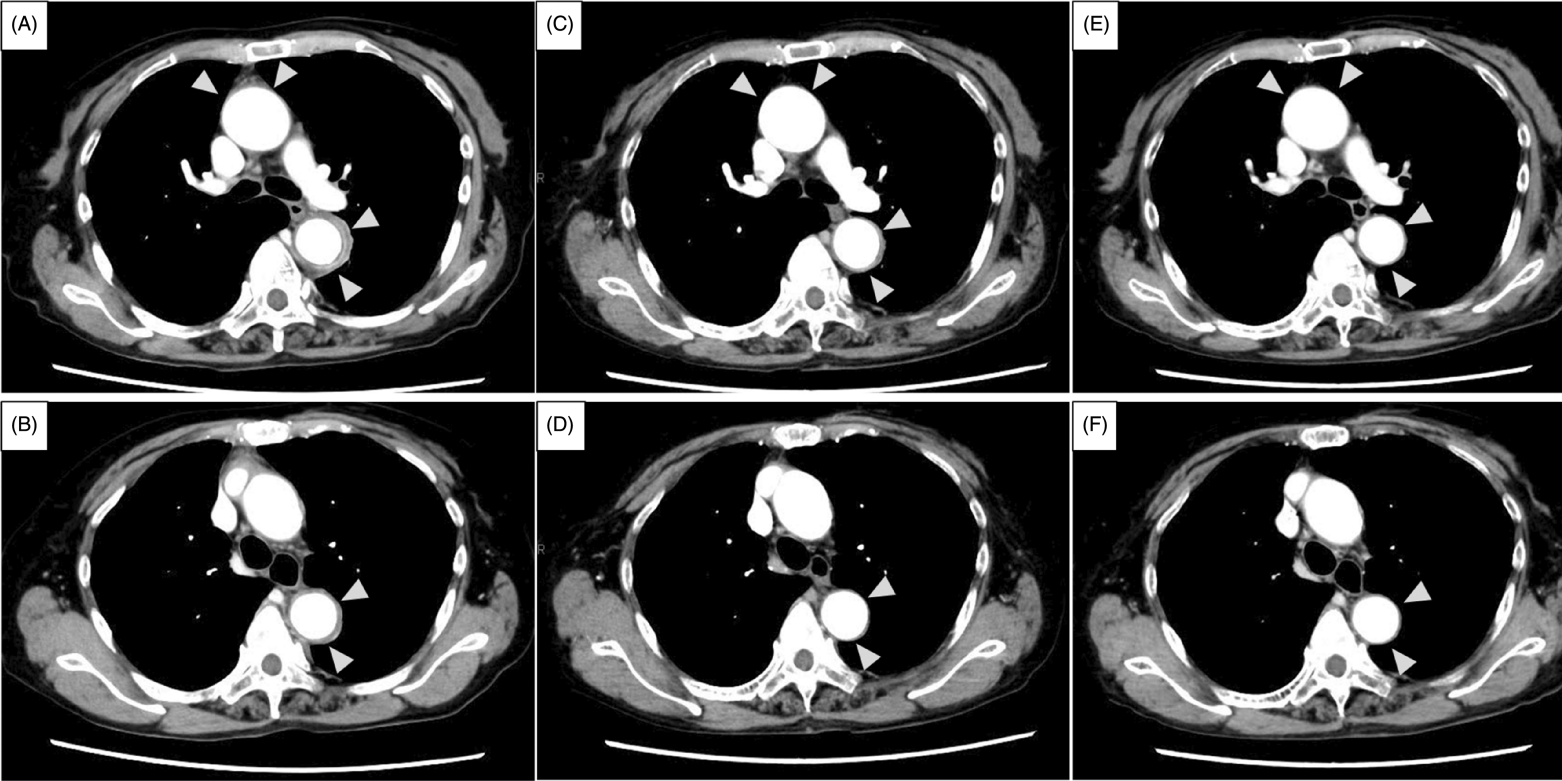
(A, B) Contrast‐enhanced computed tomography (CE‐CT) on day 15 after the first pegfilgrastim administration showing soft tissue thickening around the descending aorta and brachiocephalic artery (white arrowheads). (C, D) CE‐CT findings on day 22 after the first pegfilgrastim administration showing improvement. (E, F) CE‐CT findings on day 39 after the first pegfilgrastim administration showing complete disappearance.

She was referred to our hospital complaining of back pain since 5 days. On admission to our hospital, the patient was conscious, and her vital signs were as follows: temperature, 36.8°C; blood pressure, 168/74 mmHg; heart rate, 68 beats/min; and respiratory rate, 24 breaths/min. The oxygen saturation was 98% in room air. No abnormal findings were observed on physical examination. Results of laboratory analyses indicated the following: C‐reactive protein concentration, 23.99 mg/dL, and interleukin‐6 (IL‐6) concentration, 83.6 pg/mL (normal range, <4.0 pg/mL). Serological testing revealed negative results for antinuclear antibody (ANA), rheumatoid factor, proteinase‐3‐anti‐neutrophil cytoplasmic antibodies (PR3‐ANCA), and myeloperoxidase‐anti‐neutrophil cytoplasmic antibodies (MPO‐ANCA) (Table 1). Contrast‐enhanced CT revealed soft tissue thickening around the descending aorta and brachiocephalic artery, without aortic dissection (Figure 1A,B). These results suggested the occurrence of LVV, and the patient was admitted to our hospital for examination and treatment.

**TABLE 1:** Laboratory findings on emergent admission to our hospital.

Haematology		Biochemistry		Serology	
WBC	21,000/μL	TP	6.1 g/dL	CRP	23.9 mg/dL
Neu	87.0%	ALB	3.1 g/dL	BNP	41.1 pg/dL
Mon	4.0%	AST	46 U/L	RF	68 U/mL
Lym	8.0%	ALT	49 U/L	ANA	<×40
Eos	0.1%	ALP	199 U/L	PR3‐ANCA	<1.0 U/mL
Bas	1.0%	γ‐GTP	326 U/L	MPO‐ANCA	<1.0 U/mL
RBC	336 ×10^4^/μL	LD	232 U/L	Anti‐CCP antibody	0.6 U/mL
Hgb	9.5 g/dL	CRE	1.33 mg/dL	sIL‐2R	1325 U/mL
HCT	30.4%	UA	3.8 mg/dL	IL‐6	83.6 pg/mL
PLT	27.3 × 10^4^/μL	UN	22 mg/dL	IgG	744 mg/dL
**Coagulation**		Na	130 mmol/L	IgA	116 mg/dL
PT	11.2 sec	K	4.4 mmol/L	IgM	23 mg/dL
PT‐INR	1.01	Cl	98 mmol/L	IgE	480 mg/dL	
APTT	26.7 sec	Ca	8.8 mg/dL	IgG4	11.9 mg/dL
D‐dimer	1.8 μg/mL	T‐Bil	0.6 mg/dL	**Urinalysis**	
				Protein	(−)
				Glucose	(−)
				Ketones	(−)
				Blood	(±)

Abbreviations: ANA, antinuclear antibody; anti‐CCP antibody, anti‐cyclic citrullinated peptides antibody; BNP, brain natriuretic peptide; CRP, C‐reactive protein; IgA, immunoglobulin A; IgE, immunoglobulin E; IgG, immunoglobulin G; IgG4, immunoglobulin G4; IgM, immunoglobulin M; IL‐6, interleukin‐6; MPO‐ANCA, myeloperoxidase‐anti‐neutrophil cytoplasmic antibodies; PR3‐ANCA, proteinase‐3‐anti‐neutrophil cytoplasmic antibodies; RF, rheumatoid factor; sIL‐2R, soluble interleukin‐2 receptor.

**FIGURE 2:**
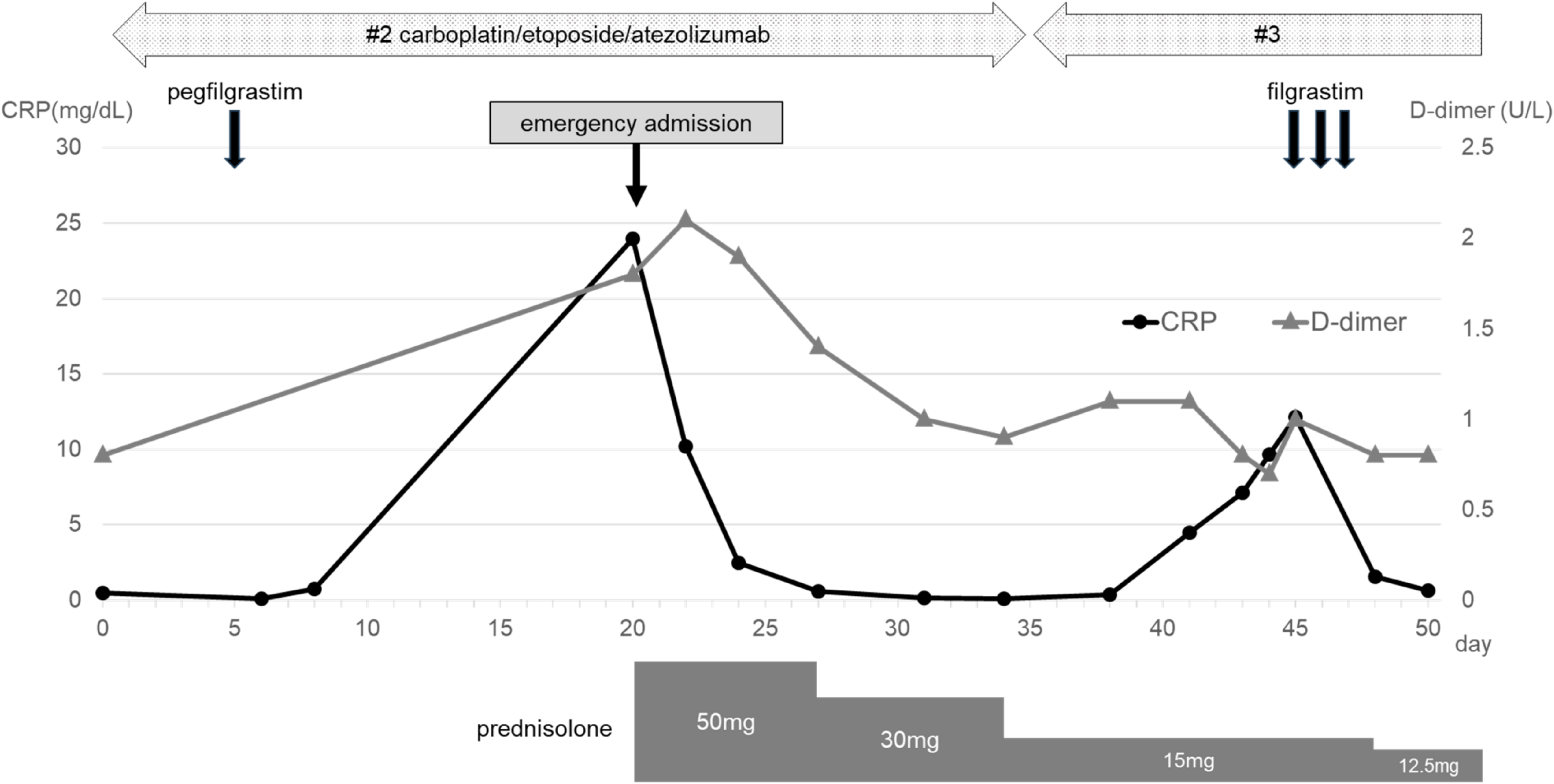
Clinical course after admission to our hospital and treatment of large vessel vasculitis. The improvement in symptoms and D‐dimer and CRP levels showed the same trend. ATZ, atezolizumab; CBDCA, carboplatin; CRP, C‐reactive protein; ETP, etoposide.

The differential diagnoses included infection, drug‐induced inflammation, and primary LVV, including ICI‐induced vasculitis due to atezolizumab‐ or pegfilgrastim‐induced LVV. The patient's clinical course is shown in Figure 2. Because an infectious aetiology had not been ruled out, she was initially treated with tazobactam piperacillin for 3 days until the blood culture results were negative. In addition, treatment with 1 mg/kg/day of prednisolone (PSL) was initiated for drug‐induced LVV. After being started on treatment, the patient demonstrated rapid improvement of back pain. The levels of the inflammatory markers were normal after 5 days of treatment. Contrast‐enhanced CT showed improvement of soft tissue thickening by day 8 (Figure 1C,D) and complete disappearance by day 25 (Figure 1E,F). PSL was tapered and discontinued after 14 weeks, with no symptom recurrence.

After the third cycle of chemotherapy, administered at the same dose as in the second cycle, the patient developed FN. Therefore, filgrastim was administered. However, the patient did not experience LVV recurrence. During the third cycle of chemotherapy, a consolidation of the right middle lobe was observed in chest CT. We speculated that the patient had developed drug‐induced pneumonia. However, it turned out to be a bacterial pneumonia associated with neutropenia, as the pneumonia was quickly resolved with antibiotics.

## DISCUSSION

LVV is caused by infectious diseases, autoimmune diseases, or drugs.^4^ Drug‐induced vasculitis is diagnosed using ultrasound, CT, magnetic resonance imaging (MRI), and [18F]‐fluorodeoxyglucose positron emission tomography (FDG‐PET).^5^ Ultrasound may be used for temporal and axillary arteries and abdominal aorta and limited value for thoracic aorta. High‐resolution MRI or FDG‐PET can be used as alternatives to ultrasound for the assessment of cranial arteries. CT may be useful for vessels throughout the body but exposes the patient to radiation. In addition, serum C‐reactive protein (CRP) level supports the diagnosis of LVV.^1^ In the present case, autoimmune diseases were excluded because serum ANA, MPO‐ANCA, and PR3‐ANCA levels were within the normal range. In addition, the patient did not meet the current diagnostic criteria of Takayasu arteritis based on the American College of Rheumatology/EULAR Classification Criteria for Takayasu Arteritis,^6^ and infectious and autoimmune diseases, such as Takayasu Arteritis, were ruled out. Thus, we considered drug‐induced LVV.

Cases of LVV induced by G‐CSF are reported to be only 0.47% of all cases of G‐CSF administration in Japan.^1^ The median time to LVV onset after G‐CSF administration is 8.0 days, with more than half of all cases occurring within 10 days.^7^ Some cases have been reported from Japan, and pegfilgrastim has been used in most of the cases ^8^ In the present case, the patient developed LVV 10 days after pegfilgrastim administration during the second cycle of chemotherapy for SCLC, which is consistent with previous reports.

As shown in Table 2, among the reported cases of vasculitis caused by pegfilgrastim in lung cancer, only the present case was treated with ICI. To the best of our knowledge, only one case of LVV after preoperative chemotherapy with pembrolizumab and subsequent chemotherapy, and pegfilgrastim for breast cancer has been reported.^9^ As shown in Table 3, three cases of vasculitis caused by ICIs in lung cancer have been reported. The patient was treated with both G‐CSF and ICI prior to the onset of LVV, and either medication might have caused LVV.

**TABLE 2:** The clinical characteristics of patients with pegfilgrastim induced large vessel vasculitis.

No.	Age	Sex	Underlying disease	G‐CSF	Time to onset LVV^a^	Anti‐cancer treatment	LVV vessels	Symptom	Treatment	Outcome	Ref.
1	67	F	NSCLC	Pegfilgrastim	8 days	N/A	Thoracic aorta and common carotid artery	General malaise and high fever	mPSL 80 mg	Resolved in 52 days	11
2	80	F	NSCLC	pegfilgrastim	11 days	Carboplatin etoposide	Common carotid artery and brachiocephalic artery	Fever and neck pain	PSL 40 mg	Resolved	15
3	61	M	Lung cancer	Pegfilgrastim	N/A	Cisplatin etoposide	Subclavian artery	N/A	N/A	N/A	16
4	71	M	LCNEC	Pegfilgrastim	10 days	Cisplatin etoposide	The aorta and left subclavian artery	Fever	PSL 40 mg	Resolved	17
Present case	75	F	SCLC	Pegfilgrastim	15 days	Carboplatin etoposide atezolizumab	Descending aorta and brachiocephalic artery	Back pain	PSL 50 mg	Resolved in 98 days	

^a^ Day after pegfilgrastim administration.

Abbreviations: F, female; G‐CSF, granulocyte colony‐stimulating factor; LCNEC, large‐cell neuroendocrine lung carcinoma; LVV, large vessel vasculitis; M, male; mPSL, methylprednisolone; N/A, not applicable; NSCLC, non‐small cell lung cancer; PSL, prednisolone; Ref, Reference; SCLC, small cell lung cancer.

**TABLE 3:** The clinical characteristics of patients with ICIs induced large vessel vasculitis.

No.	Age	Sex	Underlying disease	G‐CSF	Time to onset LVV^a^	Anti‐cancer treatment	LVV vessels	Symptom	Treatment	Outcome	Ref.
1	57	M	NSCLC	None	9 months	Nivolumab	Distal infrarenal abdominal aorta	Back pain	mPSL 60 mg	Resolved	18
2	66	M	NSCLC	None	24 months	Nivolumab	Abdominal aorta	N/A	None	Resolved in 2 months	19
3	57	M	NSCLC	None	1 months	Carboplatin pemetrexed pembrolizumab	Aortic arch	Fever	PSL 25 mg	Resolved	20
Present case	75	F	SCLC	Pegfilgrastim	1.5 months	Carboplatin etoposide atezolizumab	Descending aorta and brachiocephalic artery	Back pain	PSL 50 mg	Resolved in 2.5 months	

Abbreviations: F, female; ICIs, immune checkpoint inhibitors; LVV, large vessel vasculitis; M, male; mPSL, methylprednisolone; N/A, not applicable; NSCLC, non‐small cell lung cancer; PSL, prednisolone; Ref, Reference; SCLC, small‐cell lung cancer.

^a^ Day after initial ICIs administration.

G‐CSF increases the number of neutrophils and stimulates the production of inflammatory cytokines,^10^ which may cause inflammation due to the autoimmune mechanism of LVV. Sato et al.^11^ reported a case of G‐CSF‐induced LVV with elevated serum IL‐6 levels, suggesting that IL‐6 is involved in the development of LVV. In contrast, ICIs have been shown to cause LVV as an immune‐related adverse event (irAE).^12^, ^13^ ICI administration increases activated T‐cell infiltration into the vasculature and the levels of inflammatory cytokines, such as IL‐6, IL‐17, and interferon gamma, which may cause LVV.^14^ IL‐6 levels were elevated in the present case, suggesting that both G‐CSF and ICIs may have contributed to vasculitis.

After reviewing their contribution, Parodis et al. concluded that moderate‐to‐high doses of corticosteroids should be started immediately after the diagnosis of LVV.^2^ In this case, the patient was successfully treated with 1 mg/kg PSL, and the LVV completely resolved as evident from blood tests and imaging findings. Furthermore, LVV recurrence did not occur during PSL tapering. As shown in Figure 2, the patient continued chemotherapy but did not experience LVV after three cycles of chemotherapy and administration of filgrastim.

In conclusion, this case suggests the inclusion of LVV in differential diagnoses with nonspecific body pain when pegfilgrastim and ICIs were used in combination. With the increased use of chemotherapy with ICIs for lung cancer treatment, caution should be exercised regarding the irAEs of vasculitis when pegfilgrastim is used.

## AUTHOR CONTRIBUTIONS

Keisuke Shiraha, Yuki Takigawa and Keiichi Fujiwara wrote the manuscript, which was then reviewed by all co‐authors.

## CONFLICT OF INTEREST STATEMENT

None declared.

## ETHICS STATEMENT

The authors declare that appropriate written informed consent was obtained for the publication of this manuscript and accompanying images.

## Data Availability

The data that support the findings of this study are available from the corresponding author upon reasonable request.
